# The Consequences of COVID-19 Toward Human Growth: The Role of Traumatic Event and Coping Strategies Among Indonesian Sample

**DOI:** 10.3389/fpsyg.2021.685115

**Published:** 2021-08-18

**Authors:** Dian Veronika Sakti Kaloeti, Lusi Nur Ardhiani, Marcus Stück

**Affiliations:** ^1^Family Empowerment Center, Faculty of Psychology, Universitas Diponegoro, Semarang, Indonesia; ^2^DPFA Academy of Work and Health, Leipzig, Germany

**Keywords:** human growth, traumatic event, coping strategies, COVID-19, Indonesian

## Abstract

COVID-19 has brought a massive psychological impact on individuals' life. The current study sets a significant purpose to test the model whether post-traumatic stress and coping strategies affect stress-related growth regarding the COVID-19 event. One hundred and ninety-nine participants have participated in an online survey in the period of lockdown. The proposed hypotheses model is further tested using PLS-SEM. The first model explains a significant moderate, 46% amount of variance for stress-related growth. With gender as moderator, the second model explains a significant 29% amount of variance for stress-related growth, which is also moderate. This study shows that active coping strategies and positive affirmation significantly influence individual stress-related growth. The trauma event (COVID-19) does not significantly affect growth. Women experience trauma compared to men, besides active coping with the COVID-19 situation is higher in men than women. Using the Bio-centric perspective, having a positive connection through acceptance and awareness of the situation, self-care, and affective interaction with others would develop growth regarding traumatic situations. Further, interventions about coping skills and positive affirmations are essential to give, especially to vulnerable groups such as women.

## Introduction

COVID-19 cases were reported for the first time in December 2019 in Wuhan, China (Wang et al., 2020). Since then, cases have increased every day and peaked in late January to early February 2020 (World Health Organization, 2020a). On March 11, 2020, the World Health Organization (WHO) officially announced the Covid-19 outbreak as a global pandemic, after 118,000 confirmed cases and 4,291 reported deaths occurred in 114 countries (World Health Organization, 2020a,b). An increase in the number of cases also occurred in Indonesia. Starting from July 28, 2019, Indonesia's COVID cases reached 102,051 people identified as having positive COVID-19, 60,539 of them recovered, and 4,901 died (Gugus Tugas Penanganan COVID-19 [Task Force for the Acceleration of Handling COVID-19], 2020). Ranks first in the highest confirmed cases of contracting countries in Southeast Asia, followed by the Philippines with 63,001 cases (Kementrian Kesehatan Republik Indonesia [Ministry of the Republic of Indonesia], 2020). Based on official data presented on the Ministry of Health website on July 19, 2020, the number of positive confirmed cases has reached 13,876,441 with 593,087 deaths (4.3%), and the number of countries affected as many as 215 countries. Meanwhile, the number of positive confirmed cases in Indonesia has reached 84,882 cases, with a total of 4,016 deaths, so the percentage of fatalities is 4.7%. Indonesia ranks first in the highest confirmed cases of contracting countries in Southeast Asia, followed by the Philippines with 63,001 cases (Kementrian Kesehatan Republik Indonesia [Ministry of the Republic of Indonesia], 2020).

The disease is spreading rapidly throughout the world due to the unique nature of the COVID-19 virus, genetic diversity, highly contagious, easy to apply, and relatively unaffected by climate variations (Mackenzie and Smith, 2020). The increasing number of cases occurred very significantly globally, reaching 16,301,736 with a mortality rate of 650,069 in 216 countries in July 2020 (World Health Organization, 2020b). An increasing number of cases also occur in Indonesia. As of July 28, 2019, COVID-19 cases in Indonesia reached 102,051 people who were positively identified as having COVID, 60,539 of them recovered, and 4,901 died (Gugus Tugas Penanganan COVID-19 [Task Force for the Acceleration of Handling COVID-19], 2020). Problems arising from the presence of COVID-19 have various psychological impacts on society. Banerjee (2020) mentioned several mental health challenges experienced by the community, including panic, phobia, anxiety, sleep disorders, and dissociative symptoms.

As the number of positive confirmed cases and death rates related to COVID-19 increases, their effects on individuals and society's psychological and economic conditions also increase (Boyraz and Legros, 2020). Psychologically, the pandemic condition is scientifically proven to cause anxiety disorders, post-traumatic stress disorder, as well as to bring about depression (Bo et al., 2020; Boyraz and Legros, 2020; Ho et al., 2020; Li et al., 2020; Liu et al., 2020; Qiu et al., 2020; Shigemura et al., 2020; Wang et al., 2020). Surveys conducted weekly by the City University of New York (CUNY) since March 13, 2020, indicate that many New Yorkers experience a variety of anxiety-related to pandemics such as infected anxiety, social isolation, job loss, inability to pay bills, and so on. Most are also related to post-traumatic stress and other mental health disorders (CUNY Graduate School of Public Health and Health Policy, 2020).

Another study conducted in China involving 1,210 respondents from 194 cities showed that 16.5% of respondents reported experiencing depressive syndrome at moderate to severe levels. Meanwhile, 28.8% experienced levels of anxiety symptoms at moderate to intense levels. At the same time, 8.1% of respondents indicated that stress levels were average to severe. Based on this research, respondents of the female sex, respondents with student status, and respondents with certain physical symptoms and low health levels show higher stress levels, anxiety, and depression (Wang et al., 2020). In line with these results, research conducted on 1,115 respondents in Turkey also showed the effects that COVID-19 influenced psychological conditions, including feelings of depression, loneliness, fear of death, lack of hope, sadness, anxiety about the future, and feelings of worthlessness (Ustun, 2020).

Traumatic events can impact individual lives, such as stress levels that affect daily life (Ponnamperuma and Nicolson, 2018). The accumulation of stress levels, in the long run, will have an impact on mental health problems and the functioning of the individual. In adolescents, traumatic events impact the emergence of fear and worry about getting the label “abnormal” or different from their peers. Exposure to trauma experienced by adolescents can affect the environment's withdrawal to encourage the emergence of destructive behaviors (National Child Traumatic Stress Network, 2010). Not only that, while growing up, traumatic events can also have an impact on the process of forming self-identity (Waterman, 2020). For example, obstacles experienced by individuals in decision making. Individuals who have experienced trauma may consider or postpone the decision so that this process hinders the formation of the individual's self-identity.

Pandemic has been proven to have a traumatic effect on humans. Fear of death for both self and family is one of the reasons for the emergence of trauma in individuals during the pandemic (Murphy and Moret-Tatay, 2021; Pérez-Mengual et al., 2021). In addition, the lockdown policy creates its own stressor due to limited social activities (Burrai et al., 2020; Roma et al., 2020).

The result can be different in each individual, influenced by several factors such as age (Bonanno, 2004; Lee et al., 2007; Yip et al., 2010; Jiang et al., 2020), gender (Mak et al., 2010; Lai et al., 2020; Sun et al., 2020), as well as the presence of risk factors and protective factors (Boyraz and Legros, 2020).

Gender influences individual coping strategies (Mohiyeddini et al., 2013). Gender differences between men and women show different reactivity and responses in dealing with stressors (Verma et al., 2011). Research conducted by Deng et al. (2016) shows that men more often have intense emotional experiences while women have stronger emotional expressivity. Although men experience intense emotions, gender stereotypes allow them not to express themselves honestly (Deng et al., 2016).

Although traumatic events often harm individuals, many studies have shown that traumatic events can also broaden one's perspective, improve their ability to overcome problems, develop personal and social skills. Some changes that occur after these stressful events are often referred to as stress-related growth (Park and Fenster, 2004; Kesimci et al., 2005; Amaral et al., 2013; Macdonald, 2019). After a person experiences stress, the change that occurs is a resilient response (Zautra and Reich, 2011). The mechanism by which a person can grow after facing a stressful situation is often explained by a cognitive coping approach (Park and Fenster, 2004), where individuals can attempt to analyze the positive meaning of stressful events experienced (Solcova and Tavel, 2017). Furthermore, Stueck (2021) stated that moral priority should be given to the survival of individual living beings on pandemic based on the biocentric health management approach. There are six biocentric fields of action during and after pandemics to overcame resilient response and to protect the connection of humans namely maintainance affective communication, maintenance of lively corporeality, contact with one's own identity and inner oriented self-reflexion together with others, construction of life sense and expression of life potentials, expansion of consciousness and perception of the wholeness, and development of ecological awareness and sustainable biocentric lifestyles and attitudes (Stueck, 2021).

In addition, various literatures have tried to develop various strategies for dealing with traumatic events during a pandemic. Ramkissoon (2020) exploring the interplay of relationships between place confinement, pro-social behavior, household pro-environmental behaviors, place attachment as a multi-dimensional construct and presenting their relationships to residents' wellbeing. Further, Majeed and Ramkissoon (2020) developed and propose a conceptual framework related perceived goodness of therapeutic landscapes, health and wellness consumption, place attachment, and re-visitation. In addition, Ramkissoon (2021) discusses COVID-19 place confinement as a context to deliver body-mind medicine interventions including psycho-social, psycho-educational, relaxation, and meditation.

The current study sets a significant purpose to test the model whether post-traumatic stress and coping strategies affect stress-related growth regarding the COVID-19 event. Specifically, the study posed the following hypothesis:

1. Post-traumatic stress has a negative and significant effect on stress-related growth
2. Coping strategies have a positive and significant effect on stress-related growth
  - Depressive strategies have a negative and significant effect on stress-related growth
  - Active coping strategies have a positive and significant effect on stress-related growth
  - Self-construction strategies have a positive and significant effect on stress-related growth
  - Religiosity and search for meaning strategies have a positive and significant effect on stress-related growth
  - Wishful thinking strategies have a negative and significant effect on stress-related growth
3. Gender moderates the relationship between post-traumatic stress, and stress-related growth
4. Gender moderates the relationship coping strategies, and stress-related growth

## Materials and Methods

### The Current Study

The current study aimed to test the model of whether post-traumatic stress and coping strategies affect stress-related growth regarding the COVID-19 event. Specifically, gender moderates the relationship between post-traumatic stress, and stress-related growth. We expect post-traumatic stress experienced by individuals is determined by gender in generating stress-related growth. Variations in individual post-traumatic stress levels are determined by gender which then affects their stress-related growth. We hope that the study can provide significant information about the role of traumatic events and coping strategies to overcome the psychological impact of COVID-19 and promote human growth through a comprehensive bio-centric approach.

### Participants

We collected online questionnaires administered through an online platform with google form during the third week of the COVID-19 and stay-at-home guidance in Indonesia. One hundred nineteen participants were given research information sheets and informed consent to be signed before filling out the questionnaire. Each participant could not fill out the questionnaire multiple times due to has been given a special code. This study was conducted via electronic to prohibited face-to-face contact—the sampling method used *non-probability sampling* with *convenience sampling*. The research project to collecting data approval was obtained from the Institutional Board, to which the first author is belonging.

Based on Table 1, of the 119 participants, 103 (86.55%) were women and had a mean age of 19.94 years (range 18–≥36). Most of them were single (94.96%), undergraduate students (92.44%), and unemployed (81.51%).

**Table 1:** Data demographics (*N* = 119).

**Category**	**Total**	
	**Frequency**	**%**
**Gender**		
Male	16	13.45
Female	103	86.55
**Age (** ***Mean*** **±** ***SD)***		
18–20 (19.6 ± 0.51)	72	60.50
21–25 (22.3 ± 1.18)	40	33.61
26–30 (26.5 ± 0.70)	2	1.68
31–35 (35 ± 0)	2	1.68
≥36 (42 ± 5.29)	3	2.52
**Marital status**		
Married	5	4.20
Single	113	94.96
Cohabiting	1	0.84
**Educational status**		
Undergraduate students	110	92.44
High school	9	7.56
**Occupational status**		
Civil servants	5	4.20
Private sector workers	8	6.72
Entrepreneur	1	0.84
Part-time worker	8	6.72
Unemployed	97	81.51

### Measures

Sociodemographic information such as gender, age, level of education, and marital status was obtained using a self-report questionnaire.

*The Impact of Event Scale-Revised* (IES-R) was initially developed by Weiss (2007) to assesses the experience of post-traumatic symptoms such as intrusion (dreams about the event), avoidance (an effort to avoid reminders of the event), and hyper-arousal (feeling watchful and on guard). It comprises 22 items with participants rate on five on a five-point Likert scale ranging from 0 (not at all) to 4 (extremely) to the extent to which each item applies to their experiences during the preceding seven days. Instructions were modified such that participants are asked about the distress caused by the COVID-19 circumstances—a higher total score indicating more post-traumatic symptoms. Cronbach's alpha is reported as 0.85.

Stress-Related Growth (SRG) was measured through the 15-item (Park et al., 1996; Cohen et al., 1998). The scale is a measure of post-traumatic growth in which participants rated how much they changed due to their most stressful event by giving the response range from 0 (no changes at all) to 6 (completely changes). All items were worded in a positive direction, for example: “I learned to be nicer to others,” “I learned that there are more people who care about me than I thought.” Higher scores indicate higher levels of growth. Cronbach's alpha is reported as 0.94.

Freiburg Questionnaire of Coping with Illness (FQCI) or Freiburger Fragebogen zur Krankheitsverarbeitung (FKV) was measured through the 35-item (Muthny, 1989). The FKV is measuring coping strategies that take into five coping subscales: Depressive coping, Active problem-focused coping, Distracting and self-encouragement, Religious faith and searching for the meaning of the illness, and Denying and wishful thinking. Items can be answered on a five-point Likert-scale (from 1 = “Applies not at all”, five = “Applies very much to me”). A mean value for each subscale is computed. Higher scores represent more use of the particular coping style. Cronbach's alpha is reported as 0.83.

All questionnaires used in this study through a scale adaptation processes including forward translation, data synthesis, backward translation, expert committee review and pretesting (Heggestad et al., 2019).

### Statistical Analysis

The analysis using WarpPLS 5.0, which is a structural equation modeling (SEM) software. It employs the Partial Least Squares (PLS) method that it can refer to it as PLS-SEM. In this study, we have carried out our model in two stages: (1) Examining the measurement model's reliability and validity. It can see from the factor loading value. Indicators are considered valid if they have a factor loading value >0.5. It can be concluded that the measurement meets the convergent validity criteria (Chin, 1998); (2) Hypothesis testing by analyzing the structural model.

## Results

This study uses a convergent validity test to ensure that the indicator is a construct of the latent variable. Convergent validity can be seen from the correlation between the indicator score with the variable score. The indicator is considered valid if it has a loading factor value> 0.5. From Table 2, it can show for FKV, three items (FKV25, FKV 27, and FKV 35) were dropped. This fall indicator will subsequently not be used in the calculation of the model. There were no dropped items from IES and SRG. Also, it can be seen that the composite reliability value on the variables of post-traumatic event, coping strategies, and stress-related growth are all >0.7, which is satisfied (Chin, 1998). Further, the AVE values on all variables are >0.5. The construct validity test is to ensure that the indicator is indeed extracted from its latent variable. Thus, concluded the indicators used in this study had met discriminant validity. This shows that the variables are consistent and can be used further for hypothesis testing.

**Table 2:** The measurement model.

**Construct**	**Item**	**λ**	**Cronbach's α**	**CR**	**AVE**
IES	IES1	0.84	0.85	0.87	0.59
	IES2	0.69			
	IES3	0.64			
	IES4	0.84			
	IES5	0.54			
	IES6	0.62			
	IES7	0.54			
	IES8	0.76			
	IES9	0.51			
	IES10	0.67			
	IES11	0.55			
	IES12	0.56			
	IES13	0.58			
	IES14	0.65			
	IES15	0.54			
	IES16	0.71			
	IES17	0.73			
	IES18	0.52			
FKV	FKV1	0.69	0.83	0.84	0.54
	FKV2	0.75			
	FKV3	0.59			
	FKV4	0.72			
	FKV5	0.78			
	FKV6	0.74			
	FKV7	0.81			
	FKV8	0.85			
	FKV9	0.75			
	FKV10	0.94			
	FKV11	0.77			
	FKV12	0.81			
	FKV13	0.72			
	FKV14	0.81			
	FKV15	0.74			
	FKV16	0.76			
	FKV17	0.77			
	FKV18	0.65			
	FKV19	0.74			
	FKV20	0.69			
	FKV21	0.51			
	FKV22	0.88			
	FKV23	0.66			
	FKV24	0.64			
	FKV26	0.63			
	FKV28	0.54			
	FKV29	0.76			
	FKV30	0.76			
	FKV31	0.82			
	FKV32	0.71			
	FKV33	0.68			
	FKV34	0.73			
SRG	SRG1	0.53	0.93	0.94	0.64
	SRG2	0.68			
	SRG3	0.76			
	SRG4	0.61			
	SRG5	0.77			
	SRG6	0.78			
	SRG7	0.76			
	SRG8	0.77			
	SRG9	0.73			
	SRG10	0.81			
	SRG11	0.75			
	SRG12	0.76			
	SRG13	0.66			
	SRG14	0.73			
	SRG15	0.65			
	SRG16	0.53			
	SRG17	0.52			
	SRG18	0.67			
	SRG19	0.57			
	SRG20	0.52			
	SRG21	0.73			

### Structure Model Testing

**Figure 1:**
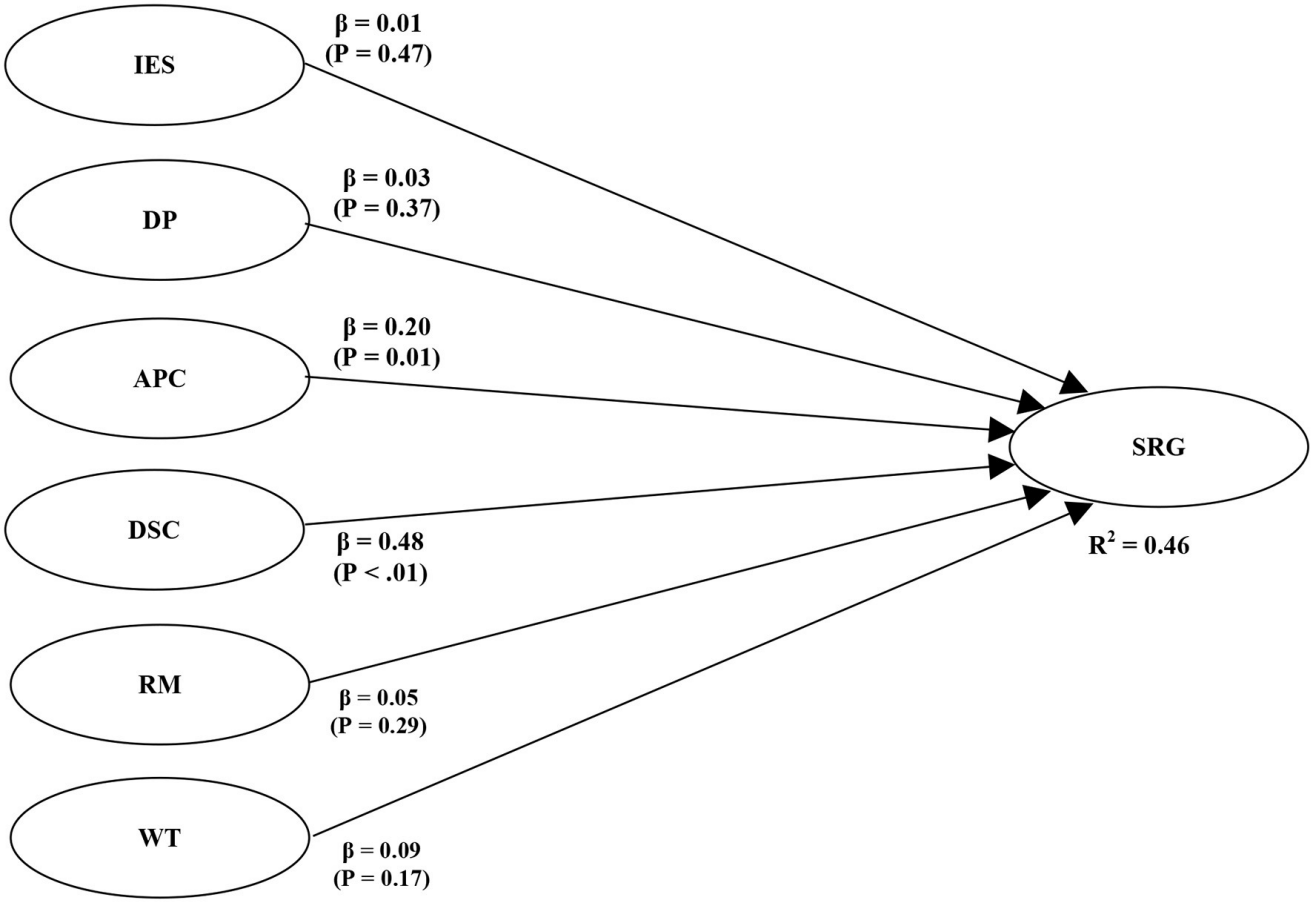
Structural model 1.

**Figure 2:**
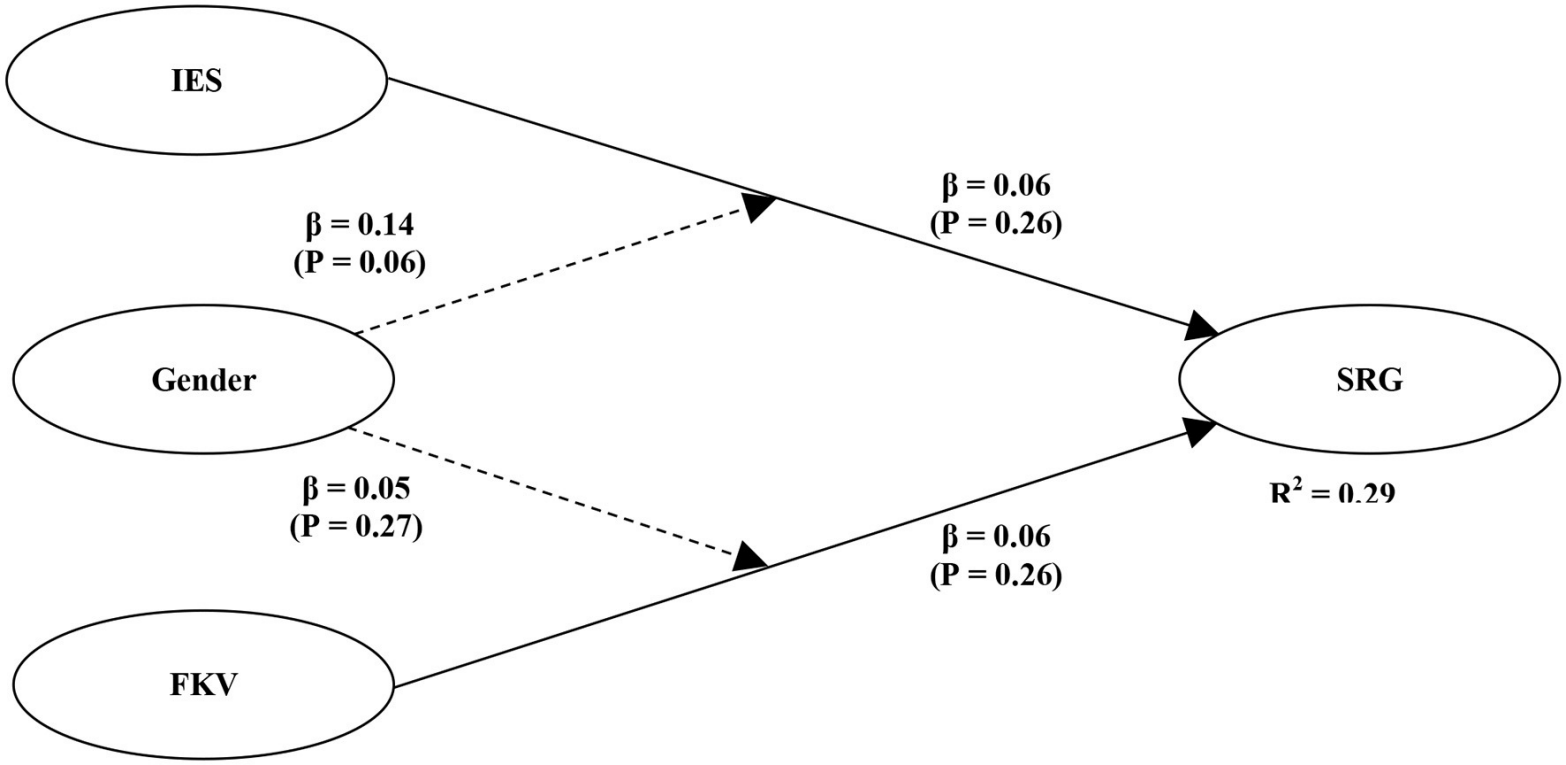
Structural model 2.

Testing the model's structure through the R Square test and Path Coefficients will be the basis for hypothesis testing. Figures 1, 2 presents the estimates obtained via PLS-SEM analysis. The model explains a significant moderate, 46% amount of variance for stress-related growth. With gender as moderator, the model presents a significant 29% amount of variance for stress-related growth, which is also moderate (Chin, 1998). It has shown the PLS path coefficients and the corresponding *p*-values for the model in Tables 3, 4. A traumatic event's impact does not have a significant effect on stress-related growth, that hypothesis 1 is rejected.

**Table 3:** Direct effect results.

	**β**	**T values**	***p*-value**	**Confidence Interval (CI)**		
IES → SRG	0.01	0.04	0.48	−0.173	0.186	Not significant
DP → SRG	−0.03	−0.33	0.37	−0.209	0.148	Not significant
APC → SRG	0.20	2.33	0.01	0.029	0.371	Significant
DSC → SRG	0.47	5.76	0.00	0.32	0.638	Significant
RM → SRG	0.07	0.72	0.24	−0.127	0.227	Not significant
WT → SRG	0.03	0.37	0.36	−0.09	0.262	Not significant

**Table 4:** Indirect effect results.

	**β**	**T values**	***p*-value**	**Confidence Interval (CI)**		
IES → SRG	−0.10	−1.16	0.13	−0.278	0.072	Not significant
FKV → SRG	0.53	6.64	0.00	0.374	0.688	Significant
Gender*IES → SRG	0.20	−1.26	0.11	−0.287	0.063	Not significant
Gender*FKV → SRG	0.47	1.47	0.07	−0.043	0.304	Not significant

In partial, the links APC → SRG (β = 0.20, *p* < 0.01), DSC → SRG (β = 0.47, *p* < 0.01) are positively related. Thus, based on beta values and corresponding *p*-values, hypotheses 2b and 2c were accepted. The event's impact, depressive processing, Religiosity, and search for meaning, and wishful thinking, do not have a significant effect in this model (see Table 3).

Next, the links FKV → SRG (β = 0.53, *p* < 0.01) are shown positively related. Hypothesis 2 is accepted.

Hypothesis 3 and 4 were tested for the moderation effect of gender on the path impact of the traumatic event and stress-related growth and coping strategies and stress-related growth (see Figure 2). Both do not significantly affect this model, so hypotheses 3 and 4 were rejected (see Table 4).

The effect size for each path model can be determined by calculating Cohen's *f* ^2^. Based on the *f* ^2^ value, the omitted construct's effect size for a particular endogenous construct can be defined such that 0.02, 0.15, and 0.35 represent small, medium, and large effects, respectively (Cohen, 1988). In Table 5, the small effect size on SRG was found in APC (0.077), RM (0.03), Gender^*^IES (0.02), and Gender^*^FKV. Further, the medium effect size on SRG was found in DSC (0.28) and FKV (0.30).

**Table 5:** Cohen's effect size.

	**Cohen's d**
IES	0.00
DP	0.01
APC	0.08
DSC	0.28
RM	0.03
WT	0.01
FKV	0.30
Gender*IES	0.02
Gender*FKV	0.05

Additional analysis was conducted to examine differences in research variables based on gender. An independent-samples *t*-test was conducted to compare all variables on gender. Based on Table 6, there was a significant difference for IES, 2.09(117), *p* < 0.00, women scored higher on traumatic event (M = 41.71, SD = 0.89) compared men (M = 36.50, SD = 2.49). Further, men indicated scored higher on active coping strategies (M = 29.98 SD = 0.30) compared women (M = 22.81, SD = 1.21); 205(117), *p* < 0.00.

**Table 6:** Independence sample *T*-test result.

**Variable**	**Gender**	**N**	**M**	**SD**	**t(df)**	***p***
IES	Women		41.71	0.89	2.09(117)	0.03
	Men		36.50	2.49		
DP	Women		15.59	1.11	0.16(117)	0.80
	Men		15.38	4.77		
APC	Women		29.98	0.30	2.05(117)	0.00
	Men		22.81	1.21		
DSC	Women		33.85	0.41	−0.13(117)	0.89
	Men		34.00	1.18		
RM	Women		34.20	0.44	1.84(117)	0.07
	Men		31.81	1.70		
WT	Women		6.48	0.18	−0.98(117)	0.06
	Men		7.00	0.71		
FKV	Women		111.11	1.11	0.03(117)	0.97
	Men		111	4.77		
SRG	Women		101.22	1.32	1.97(117)	0.06
	Men		109.25	2.81		

## Discussion

This study found that participants were exposed to trauma from the emergence of the COVID-19 outbreak in Indonesia. Inconsistent with some other studies (Siqveland et al., 2015; Zieba et al., 2019), trauma condition scores do not significantly affect the stress-related growth experienced by individuals. This is because the emergence of growth requires gradual cognitive processes and perceived traumatic experiences.

Furthermore, Brooks et al. (2016) revealed that strategic social support and coping stress allow trauma or life burdens experienced not to predict growth in participants. In line with this opinion, this study found that participants' coping significantly affected the emergence of stress-related growth. Machado et al. (2020) stated that habitual use of coping strategies is important part of the treatment in posttraumatic stress symptoms. Moreover, specifically, Girma et al. (2021) stated about Covid-19 Pandemic-Related Stress that coping strategies significantly helped patient with chronic disease. Coping strategies are significant predictors for mental health measures on traumatic events due to could improve positive thinking (Budimir et al., 2021) and resilience (Gori et al., 2021). Stress's positive or negative effects depend on coping strategies that individuals have in solving problems (Park et al., 2009). An experimental study conducted on 94 students showed that individuals' coping activities could increase individual growth, including generating positive thoughts (Park and Fenster, 2004). Individuals who use positive coping strategies tend to report high growth levels (Bi et al., 2016). Growth will impact improving personal skills and interpersonal relationships (Kesimci et al., 2005; Popa and Podea, 2013).

The coping process mechanism has an impact on one's growth after passing through an adverse experience. Research conducted by Wild and Paivio (2003) of 193 student participants related to post-traumatic growth (PTG) shows the results that active coping and subjective well-being affect the post-traumatic growth (PTG) subject. Meanwhile, research conducted on 256 people who survived the 2010 Haiti earthquake showed a significant positive correlation between post-traumatic growth (PTG) and active coping (Mesidor and Sly, 2019). This is also in line with Park and Fenster's research on 94 Psychology students who showed the results that both coping strategies and cognitive processes play a role in the occurrence of growth after stressful growth experiences. This study states that individuals' efforts to overcome their stressors determine the growth experienced (Park and Fenster, 2004). Supporting this statement, Amaral, in his research results, revealed that coping strategies when facing problems influence the increase in post-traumatic growth subjects (Amaral et al., 2013).

One of the positive coping strategies is self-encouragement and self-affirmation. Self-affirmation affects the openness and behavior of individuals (Stapel and van der Linde, 2011; Main and Dillard, 2012). Research conducted by Creswell et al. (2013) shows that self-affirmation can protect individuals from the adverse effects experienced by stress-related problem-solving. This research shows that individuals' chronic stress can interfere with their problem-solving performance, and self-affirmation can improve problem-solving performance under pressure. Self-affirmation protects individuals from the adverse effects of stress on problem-solving performance, even in situations of acute or ongoing stress. Self-affirmation can provide long-term influence on one's ability to grow and adapt to deal with problems that cause anxiety (Cohen and Sherman, 2014). In a study conducted on 80 student participants, self-affirmation is known to encourage someone to be more creative so they can find insight when facing stressful and stressful events in life. Furthermore, Self-affirmation can improve one's mental health condition (Creswell et al., 2013).

Furthermore, the women in this study were more vulnerable to trauma situations. This is supported by research that women are more sensitive to traumatic experiences (Stroebe et al., 2005; Rzeszutek et al., 2017). Dell'Osso et al. (2012) also reported that women significantly higher PTSD prevalence rates and post-traumatic spectrum symptoms than men. In addition, Gentry et al. (2007) reported that women has higher in overall perceived stress levels, but there was no difference in the experienced social stressors and health stressors between genders. The sensitivity of women causes the emergence of self-brooding rumination) and a lack of thinking about self-reflective rumination (Lund et al., 2010). Furthermore, Stroebe et al. (2005) allude to the tendency of men to be oriented toward cognitive activity, so that this allows men to be more adaptive in dealing with trauma and lead to growth.

This study shows that active coping strategies and positive affirmation significantly influence individual stress-related growth. The trauma event (COVID-19) does not significantly affect growth. We assumed it because the data collection process carried out when COVID-19 has just occurred so that the construction process of the event's meaning has not been produced.

Women experience trauma compared to men, besides active coping with the COVID-19 situation is higher in men than women. Herren et al. (2021) also shown that there are differences in coping strategies between men and women. Women are more likely than men to report negative affective outcomes from emotion suppression (Herren et al., 2021). Further, Gentry et al. (2007) showed that women were more likely to use adaptive coping strategies, whereas men were more likely to use maladaptive and avoidance coping strategies. Interestingly, the religious matter is not yet significant. We assumed when in the initial phase of the pandemic, individuals are still in a state of shock, so connecting pandemics with faithful things still difficult.

This study illustrates the importance of active coping and self-affirmation in dealing with adverse situations such as COVID-19. Interventions about coping skills and positive affirmations are essential for vulnerable groups such as the women population. The number of male participants are not equal to the number of women is a limitation in this study.

Future studies will undoubtedly benefit from longitudinal designs using more diverse samples. We plan to continue this research to make repeated measurements of trauma and post-traumatic conditions, explore more deeply growth factors regarding COVID-19, and broaden the study participants' demographic characteristics.

## Data Availability Statement

The raw data supporting the conclusions of this article will be made available by the authors, without undue reservation.

## Ethics Statement

The studies involving human participants were reviewed and approved by Faculty of Psychology, Diponegoro University. The patients/participants provided their written informed consent to participate in this study.

## Author Contributions

DK: research design and manuscript writing, modeling, data collection, and data processing. MS: research design and manuscript writing. LA: manuscript writing. All authors contributed to the article and approved the submitted version.

## Conflict of Interest

The authors declare that the research was conducted in the absence of any commercial or financial relationships that could be construed as a potential conflict of interest.

## Publisher's Note

All claims expressed in this article are solely those of the authors and do not necessarily represent those of their affiliated organizations, or those of the publisher, the editors and the reviewers. Any product that may be evaluated in this article, or claim that may be made by its manufacturer, is not guaranteed or endorsed by the publisher.
